# Emergency anaphylaxis protocols: A cross-sectional analysis of general practice surgeries and pharmacies in both the urban and rural setting in Ireland

**DOI:** 10.1080/13814788.2018.1480717

**Published:** 2018-09-26

**Authors:** Hannah O’Brien, David Mc Conaghy, Declan Brennan, Sarah Meaney

**Affiliations:** a Department of Palliative Medicine, Marymount University Hospital and Hospice, Cork, Ireland;; b General Practitioner, GP Trainer and Occupational Health Consultant, Sallins, Co. Kildare, Ireland;; c General Practitioner and Training Programme Director, Mullingar, Co.Westmeath, Ireland;; d National Perinatal Epidemiology Centre, Cork University Maternity Hospital, Cork, Ireland

**Keywords:** Anaphylaxis, protocols, kits, general practitioner, pharmacist

## Abstract

**Background:** The incidence of anaphylaxis appears to be increasing worldwide with cases in the community outnumbering those in the hospital setting. General practice (GP) surgeries and pharmacies, based in the community, are often the first point of contact for many patients suffering from anaphylaxis.

**Objectives:** To determine if studied GP surgeries and pharmacies have an anaphylaxis protocol on site and have access to an anaphylaxis kit; to explore GP’s and pharmacists’ personal experiences with management of anaphylaxis.

**Methods:** A cross-sectional, questionnaire-based study was performed examining anaphylaxis protocols in a sample of general practices and pharmacies from some counties in Ireland. This consisted of a sample from rural and urban settings. The study commenced in October 2014.

**Results:** Nineteen of 24 GPs (79%) and 9 (29%) pharmacies had an anaphylaxis protocol (*P* < 0.001). Twenty-four (100%) GP practices and 12 pharmacies (39%) surveyed had an anaphylaxis kit on site. Twelve GPs (50%) had treated a patient with anaphylaxis in the surgery while 8 (33%) had treated a patient in the community. One pharmacist (3%) had witnessed anaphylaxis in practice. Two pharmacies and one GP had been contacted by local businesses to alert them to a case of anaphylaxis.

**Conclusion:** In contrast to national and international guidelines only 79% of GPs and 29% of pharmacies in this study from Ireland had an anaphylaxis protocol onsite.

KEY MESSAGES

- Seventy-nine percent of GP practices had an anaphylaxis protocol in comparison with 29% of pharmacies.
- Fifty percent of general practitioners (GPs) had managed anaphylaxis in their practice, 33% attended a case in the community.
- National and international guidelines emphasise the importance of having an emergency protocol for anaphylaxis management.

## Introduction

The incidence and prevalence of anaphylaxis appears to be increasing with cases in the community outnumbering those in the hospital setting [1–6]. Estimated incidence rates vary between 1.5–7.9 to 590 per 100,000 person years in Europe [7,8]. The prevalence has been estimated at 0.3% of the European population to lifetime prevalence of 50 to 2,000 per 100,000 persons or 0.05 – 2% of the population [3,9]. It has been estimated that 20 to 30 deaths attributable to anaphylaxis occur each year in the UK [4]. The first ever reaction resulted in fatal anaphylaxis in two-thirds of cases [4]. The World Allergy Organisation and the American Academy of Allergy and Immunology (AAAAI)/American College of Allergy, Asthma and Immunology (ACAAI) guidelines emphasise the importance of having an emergency protocol for the management of anaphylaxis [1,10–12].

Evidence suggests that late administration of intramuscular adrenaline as first-line treatment for anaphylaxis is associated with worse outcomes [8,13]. Auto-injectors are prescribed to those who are considered to be at high risk of anaphylaxis and in whom allergens cannot easily be avoided e.g. food-induced, insect venom sensitivity or idiopathic reactions [2,4]. It was estimated in studies that of those prescribed auto-injectors, only 50–75% carried them and just 30-40% could accurately demonstrate their use [4,13].

The Irish Health Service Executive (HSE) Immunisation Guidelines contain an anaphylaxis protocol, ‘Anaphylaxis Treatment in the Communit’ with a suggested anaphylaxis kit [14]. The Irish College of general practitioners (ICGP) refers to the online Immunisation Guidelines Anaphylaxis Protocol [14]. The Pharmaceutical Society of Ireland (PSI) Seasonal Influenza Vaccination Training Programme for pharmacists registered in Ireland for the 2014 – 2015 ‘Flu season includes CPR and anaphylaxis management training [15]. It recommends that pharmacists are familiar with the most recent versions of both the National Immunisation Advisory Committee (NIAC) and HSE National Immunisation Office (NIO) national guidance documents on immunisations [14,15].

It has been demonstrated in Ireland that a patient may attend their nearest pharmacy when suffering from acute anaphylaxis [16]. This was evident in the tragic case that occurred on O’Connell Street in Dublin on 18 December 2013, where a patient inadvertently ingested peanut [16]. Community Pharmacy Scotland has launched an initiative the ‘Orange Cross’ scheme whereby pharmacies that display this are trained and prepared to provide help to patients suffering from anaphylaxis [17].

The motivation for this study was the increasing incidence of anaphylaxis in the community and the role that both general practitioners and Pharmacists play. The purpose of this study was to determine if studied GP surgeries and pharmacies have an anaphylaxis protocol on site, have access to an anaphylaxis kit and to explore GPs and pharmacists’ personal experiences with management of anaphylaxis.

## Methods

### Study Design

A cross-sectional study of general practitioners (GPs) and Community Pharmacists was designed with samples from both urban and rural practices in Ireland. A research planning meeting was held and a total sample size of 100 professionals, 50 of whom were GPs and 50 who were Pharmacists was decided upon. Due to the small number of participants, this study was not powered. Questionnaires were designed and posted on 11 October 2014. Responses were received in the practice where they were included in the daily post over the months of October to December 2014.

### Ethics

Ethical approval was not required as the information received was anonymised and this study would not involve any intervention in patient care.

### Selection of study subjects

Fifty GP practices were selected from the Irish Medical Directory (IMD). Twenty-five were urban practices from the cities of Dublin, Cork, Limerick and Galway. Twenty-five were rural practices included from the counties of Kildare, Cork, Limerick and Galway. These were taken beginning from the start of the alphabet in the Irish Medical Directory (IMD) with no two consecutive practices residing in the same postcode or town. Fifty pharmacies were selected from the IMD in the same fashion. Pharmacies based within a hospital were excluded as patients did not have direct access and supply of adrenaline pens to patients was not performed.

### Data collection

Two structured questionnaires – one for GPs, the other for pharmacists – were designed based on a literature review and discussion with peers. They were drafted and piloted in September 2014 among a group of GP Registrars, GPs and Pharmacists. The questionnaire was revised and amendments made (Supplementary material, available online). The questionnaire included demographics of the professionals, demographics of their practices, and explored GP and pharmacists’ experiences and preparedness in the management of anaphylaxis. The self-reported questionnaires, enclosed with a cover letter, were posted inviting the party to participate in the study. A stamped addressed envelope to facilitate the questionnaires return was included. This was an anonymous study and as such questionnaires were not numbered.

### Data analysis

A Microsoft Excel spreadsheet was created and the data was input into this application. The majority of the questions were categorical with some questions containing spaces for comments or expansion on the answers. The results were examined using descriptive means. The results were then exported to the IBM SPSS statistical program where they were examined using Chi-squared analysis looking for statistical significance and confidence intervals were calculated.

## Results

One hundred questionnaires were distributed, fifty to GPs and fifty to pharmacists. Twenty-five were sent to GP and pharmacy practices in an urban setting and a further 25 to practices in a rural setting. A total of 25 GP and 31 Pharmacists completed questionnaire responses were received. This gave a response rate of 55%. Due to a defined study period, a second wave of questionnaires were not sent out at that time. Table 1 illustrates the demographics of respondents.

### Anaphylaxis protocols and anaphylaxis kits

GPs (*N* = 19) (79%; CI 62–97) were more likely to have a protocol in place than pharmacists (*N* = 9) (29%; CI: 12–46) (*P* < 0.001; CI: 2.6-32.3). All 24 GP practices had an anaphylaxis kit on site compared with 12 pharmacies (39%; CI: 21-57) (*P* < 0.001). Two pharmacies commented that they had adrenaline on site for flu patients. Employees knew where the anaphylaxis kit was in twenty (83%) GP practices. Of the 12 pharmacies with anaphylaxis kits, everyone knew the kits’ location in eight (67%).

The anaphylaxis kit was checked regularly in 23 (96%) of GP practices and in 11 (92%) of pharmacies surveyed. Twenty GP practices (83%) had a designated person to check the kit compared to seven pharmacies (58%) (*P* = 0.056). There was a log to check anaphylaxis kits in five (21%) of GP practices and five (42%) of those pharmacies with kits. Sixteen GP participants (67%) had an anaphylaxis kit in their doctors’ bag. Table 2 shows that 88% of GP anaphylaxis kits contained adrenaline, chlorphenamine and steroids. Adrenaline was in ampoule form in 16 of these kits (67%) while six contained the auto-injector form (25%). Ten (83%) pharmacist anaphylaxis kits contained adrenaline alone. One pharmacy kit contained adrenaline, steroids and chlorphenamine (3%) while another contained adrenaline and chlorphenamine (3%). All 12 pharmacies with anaphylaxis kits contained the auto-injector preparation of adrenaline.

**Table 1.** Demographics.

Demographics of respondents	GPs *N* = 24*	Pharmacists *N* = 31
Sex		
Male respondents	16 (63%)	16 (52%)
Female respondents	9 (37%)	15 (48%)
Type of practice		
Urban Practice	9 (37%)	14 (45%)
Rural Practice	4 (16%)	7 (23%)
Mixed (urban and rural)	10 (41%)	8 (26%)
Post-graduate experience	23 (96%)	16 (52%)
GP Trainer	3 (12%)	N/A

* 25 GPs returned questionnaires, of those 25 responses received, 24 were currently working in general practice giving a total of 24 respondents.

**Table 2.** Anaphylaxis kit contents.

Contents of anaphylaxis kit	GPs *N* = 24	Pharmacists *N* = 12*
Anaphylaxis kit on site	24 (100%)	12 (39%)
Adrenaline, chlorphenamine and steroids	21 (88%)	1 (8%)
Adrenaline and chlorphenamine	1 (4%)	1 (8%)
Adrenaline alone	0	10 (83%)
Not known	1 (4%)	0
No response	1 (4%)	0
Adrenaline ampoule	16 (67%)	0
Adrenaline auto-injector	6 (25%)	12 (100%)
Not applicable (No anaphylaxis kit on site)	0	19 (61%)

* Of those pharmacies surveyed, 12 had an anaphylaxis kit on site)

Most GPs who responded to the question (*n* = 21, 91%) had steroids in the kit compared to 1 (8%) pharmacist (*P* < 0.001). The majority of GPs that responded (*N* = 22, 95%) had chlorphenamine in the kit compared to 2 (17%) pharmacists (*P* < 0.001).

Six GP practices (25%) in this study were available to businesses or institutions in a case of anaphylaxis. Twenty-nine of the pharmacies had access to a GP or hospital in a case of anaphylaxis (94%).

#### Auto-injector administration

To date, 23 pharmacists had received training in the administration of an auto-injector (74%). Twenty-five pharmacists (81%) surveyed would be interested in formal training of administration of an auto-injector, 17 of whom had previously undergone training. Of note, all five pharmacists who had not had emergency response training would be interested, two who had previous instruction with an auto-injector.

#### Emergency response training and provision of guidelines

General practitioner emergency response training varied. Twenty GPs (83%) had previous training while 1 (4%) never had emergency response training. Three GPs did not provide an answer (13%). Fourteen GPs had attended training in the past two years (58%), six in the preceding seven years (25%). Twenty-six (84%) pharmacists had received emergency response training, 3 of whom as part of vaccination training. Five respondents had not previously received training (16%).

Eighty-seven percent of GPs and 97% of pharmacists felt that the ICGP and PSI respectively should produce and distribute guidelines. One GP commented that there are guidelines distributed through the small group network.

#### Awareness and experience of anaphylaxis cases

**Figure 1.**
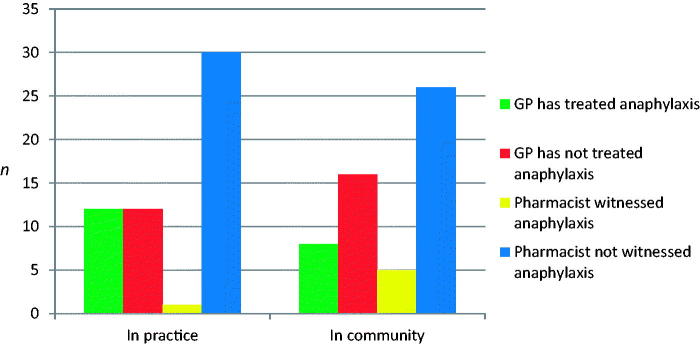
General practitioner (GP) (*N*) and pharmacists (*N*) experience with anaphylaxis in both practice and the community.

Eight, (33%; CI: 14–56) GPs and 14 (45%; CI: 27–64) pharmacists were aware of a recent case of anaphylaxis. There were five responses with comments including two pharmacists who referenced the case in O’Connell Street, Dublin. Twelve GPs (50%) had treated a patient with anaphylaxis in the surgery while 8 (33%) GPs had treated a patient with anaphylaxis in the community. One (4%; CI: 4–13) of the GPs surveyed had been called by a local business to attend a case of anaphylaxis (Figure 1). One pharmacist (3%) had witnessed anaphylaxis in practice. Sixteen percent of pharmacists had witnessed anaphylaxis in the community. Two pharmacists (7%; CI: 3–16) had been called by a local business to alert them about a case of anaphylaxis (Figure 1).

## Discussion

### Main Findings

This study found that 12 (50%) GPs had treated a case of anaphylaxis in surgery, eight (33%) had managed a case in the community, and one (4%) had been called by a local business to attend to a patient. One (3%) pharmacist had experienced a case of anaphylaxis in the pharmacy, five (16%) had witnessed anaphylaxis in the community and two (6%) had been called by a local business to alert them to a case of anaphylaxis. Although this is a small sample, it demonstrates that patients may attend the nearest general practice or pharmacy in cases of anaphylaxis in line with previous studies [16]. Nineteen (79%) GP surgeries had an anaphylaxis protocol on site and all of these practices checked their anaphylaxis kits regularly. There was an approved protocol in just nine (29%) pharmacies. Anaphylaxis kit contents differed in both GP practices and pharmacies, which may suggest a variation in protocols followed. Emergency response training was variable; basic life support (BLS) and advanced cardiac life support (ACLS), which both include CPR, have a 2-year certification period and it is advisable that GPs and pharmacists hold up to date emergency response training. Seventy-four percent of pharmacists surveyed had received training in the administration of an auto-injector. Despite this, 81% of the pharmacists surveyed would be interested in formal training, demonstrating the need for reinforcement and repetition of training. Most GPs and pharmacists felt that the ICGP and PSI respectively should produce and distribute guidelines. This may help to improve preparedness in the practices studied.

#### Comparison with other studies

A study performed to evaluate how community pharmacists manage patients with anaphylaxis, found that ‘pharmacists demonstrated reasonable knowledge of anaphylaxis symptoms and emergency care but had poor epinephrine auto-injector technique and rarely discussed anaphylaxis action plans' [17]. Participants in this small study demonstrated a clear interest in further training in administration of an auto-injector. Another Australian based study looked at community pharmacists’ responses to emergency situations, including administration of adrenaline, found that a need for pharmacists ‘to be trained to respond to a medical emergency, with specific reference to the administration of certain S3 medicines’ including adrenaline. Both studies recommend training and protocols be developed for practicing community pharmacists [17,18].

Both national and international guidelines recommend that protocols are on kept onsite to manage this emergency situation [1,10–12,14,15].

#### Strengths and limitations

This appears to be the first study looking at anaphylaxis protocols in GP surgeries and pharmacies in Ireland. There was a representation of both urban and rural settings in Ireland. There were a small number of participants, which may affect the external validity of this study. The response rate may have been improved by sending further waves of questionnaires or following up by a phone call in addition to gaining an understanding why a large number of professionals declined to participate in this study. As this research was carried out as part of a training programme, with a defined study period, a second wave of questionnaires was not sent out at that time. The pharmacists’ questionnaire did not include a question regarding administration of the flu vaccination on their premises, which may correlate with those having an anaphylaxis protocol or kit on site. The questionnaire did not specify what ‘community’ referred to and this could have meant a variety of locations.

### Implications

In this study, a majority of GPs had a protocol and all had an anaphylaxis kit on site in comparison to a minority of pharmacies. This study did not examine why practices surveyed did not have a protocol in place, or to which protocol they referred, and this would be important to elucidate in future work. Recognition of anaphylaxis may determine whether a protocol is activated and healthcare professionals’ confidence in identifying anaphylaxis would be a valuable study. Investigating whether GPs have received formal training in the use of an auto-injector, their confidence in demonstrating its use to patients and whether they would like to receive training in this area is another vital area for future work.

## Conclusion

In contrast to national and international guidelines, only 79% of GPs and 29% of pharmacies in this study from Ireland had an anaphylaxis protocol on site.

## Supplementary Material

Supplemental Material
