# Supplementary figures and images for: Emergency anaphylaxis protocols: A cross-sectional analysis of general practice surgeries and pharmacies in both the urban and rural setting in Ireland
Source: Eur J Gen Pract. 2018 Sep 26;24(1):223–8. doi: 10.1080/13814788.2018.1480717 (PMC6161615; doi:10.1080/13814788.2018.1480717)

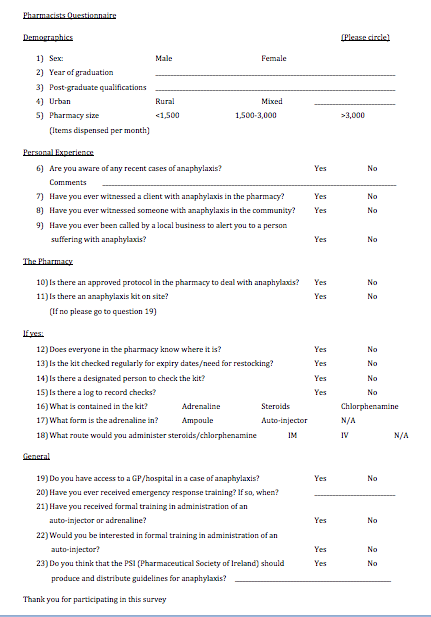


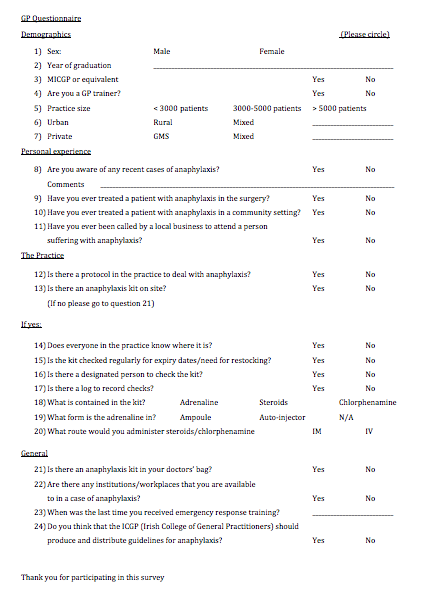

Supplement: Supplemental Material [file IGEN_A_1480717_SM0711.docx]
